# Pupillary Constriction and Dilatation as a Window into Brainstem and Autonomic Integrity

**DOI:** 10.21315/mjms-01-2026-079

**Published:** 2026-02-28

**Authors:** Surin Etican, Zamzuri Idris, Diana Noma Fitzrol

**Affiliations:** 1 Department of Neurosciences, School of Medical Sciences, Universiti Sains Malaysia, Health Campus, Kelantan, Malaysia; 2 Brain and Behaviour Cluster, School of Medical Sciences, Universiti Sains Malaysia, Health Campus, Kelantan, Malaysia; 3 Department of Neurosciences and Brain Behaviour Cluster, Universiti Sains Malaysia Specialist Hospital, Universiti Sains Malaysia, Health Campus, Kelantan, Malaysia

Dear Editor,

We read with interest the recent letter to the editor addressing patterns of upward, lateral, and downward brain herniation and their correlation with clinical findings in acute intracranial pathologies (1). In that work, the authors discussed relevant neuroanatomical considerations, including the process of intracranial herniation and its relationship to pupillary size and light responsiveness. We wish to elaborate further on pupillary changes, with particular emphasis on variations in size and reactivity and their correlation with the stages of rostrocaudal herniation.

The pupils act as the dynamic optical aperture of the visual system, continuously modulating retinal illumination and visual performance in response to environmental light, cognitive demand, and autonomic tone (2, 3). In wellness, pupillary constriction (miosis) and dilatation (mydriasis) are physiological, symmetric, and reversible, reflecting intact parasympathetic–sympathetic balance and preserved supranuclear control (2). In contrast, in illness, miosis and mydriasis often differ qualitatively—becoming fixed, asymmetric, exaggerated, or dissociated from visual stimuli— thereby signalling disruption along the afferent visual pathways, brainstem nuclei, autonomic circuits, or their cortical modulation (2). Thus, while normal pupillary size fluctuates adaptively, pathological pupillary changes tend to persist or behave incongruently, transforming a physiological aperture into a clinically meaningful neurological sign (2).

## Relevant Neuroanatomy

The pupil is the central opening of the iris, functioning as the adjustable aperture of the eye that regulates the quantity of light reaching the retina (3). Unlike a passive opening, pupillary size is actively controlled by specialised smooth muscles within the iris, allowing continuous and precise modulation in response to physiological and pathological states (2, 3).

**Figure 1:**
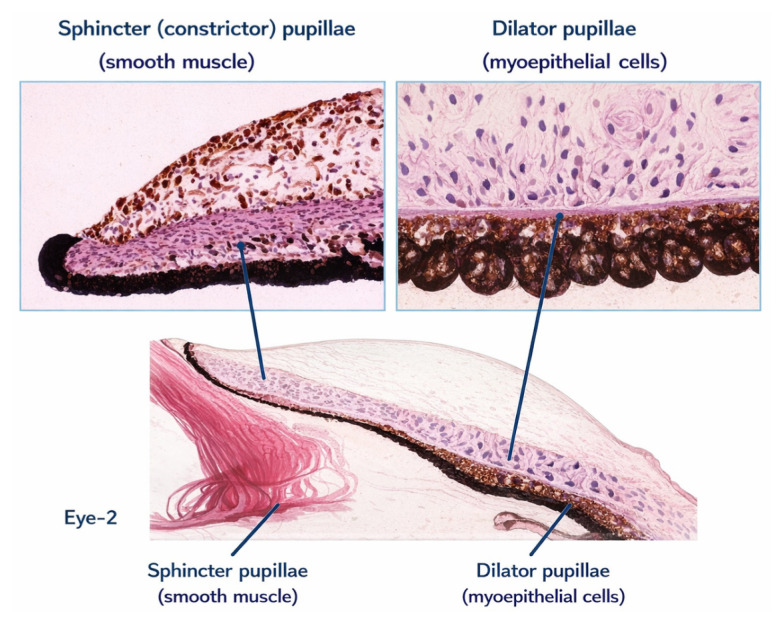
Location of sphincter and dilator pupillae muscle in relation to the pupils

**Figure 2:**
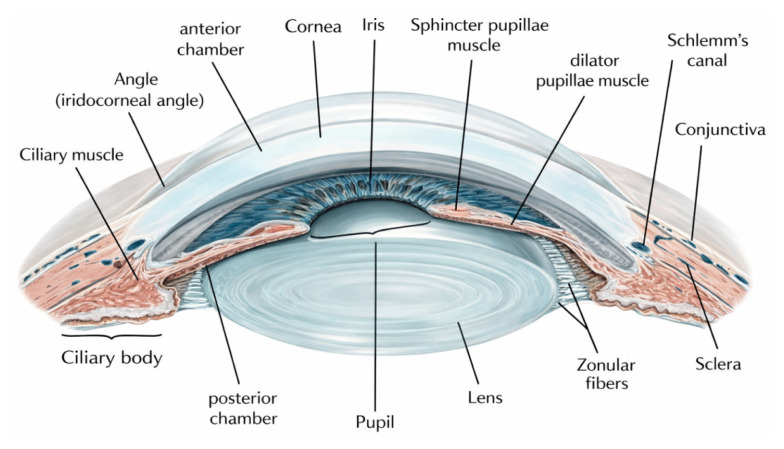
Cross-section of the human eye

Two distinct smooth muscles govern pupillary diameter: the sphincter pupillae and the dilator pupillae (Figure 1) (2, 3). These muscles differ in their anatomical arrangement, direction of action, and functional contribution to pupillary dynamics. The coordinated activity of these two iris muscles determines pupillary size at any given moment, forming the anatomical foundation upon which neural control mechanisms act in both health and disease (Figure 2) (2, 3).

### Sphincter Pupillae

The sphincter pupillae is a thin, circumferential band of smooth muscle located at the pupillary margin of the iris. Contraction of this muscle narrows the pupillary aperture, resulting in miosis. Its circular orientation permits uniform reduction in pupillary diameter, contributing to regulation of retinal illumination, enhancement of depth of focus, and protection of the retina from excessive light exposure (3).

### Dilator Pupillae

The dilator pupillae consists of radially arranged smooth muscle fibres extending from the peripheral iris toward the pupillary margin. Contraction of these fibres increases pupillary diameter, producing mydriasis. This radial configuration enables rapid pupillary enlargement, facilitating improved retinal illumination under low-light conditions and during states requiring heightened visual awareness (3).

### Parasympathetic Pathway Mediating the Pupillary Light Reflex

Light entering the eye is focused onto the retina, where phototransduction occurs within rods and cones. Signals are transmitted via bipolar cells to retinal ganglion cells, whose axons form the optic nerve. In adult humans, the optic nerve measures approximately 45 to 50 mm in total length, comprising an intraocular segment (approximately 1 mm), intraorbital segment (approximately 25 to 30 mm), intracanalicular segment (approximately 6 to 10 mm), and intracranial segment (approximately 10 mm). These measurements are derived from gross anatomical and radiologic studies of the human optic nerve and are summarised in standard neuroanatomical references (2, 4). The fibres subserving the pupillary light reflex course within the optic nerve and optic tract, but diverge from the geniculostriate visual pathway prior to synapsing in the lateral geniculate body (2).

**Figure 3:**
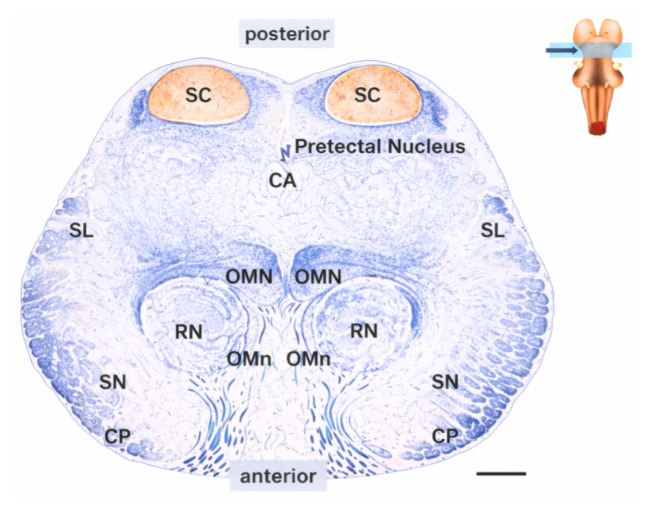
Cross-section of the midbrain showing the pretectal nucleus

From the optic tract, afferent pupillary fibres synapse in the pretectal nuclei of the dorsal midbrain at the level of the superior colliculus. Neurons within each pretectal nucleus project bilaterally to the Edinger–Westphal (EW) nuclei via the posterior commissural region. Although the precise length of the pretectal– EW internuclear connections has not been quantified in humans, their bilateral organisation is consistently demonstrated and forms the anatomical basis of the consensual light reflex (Figure 3) (3, 4).

**Figure 4:**
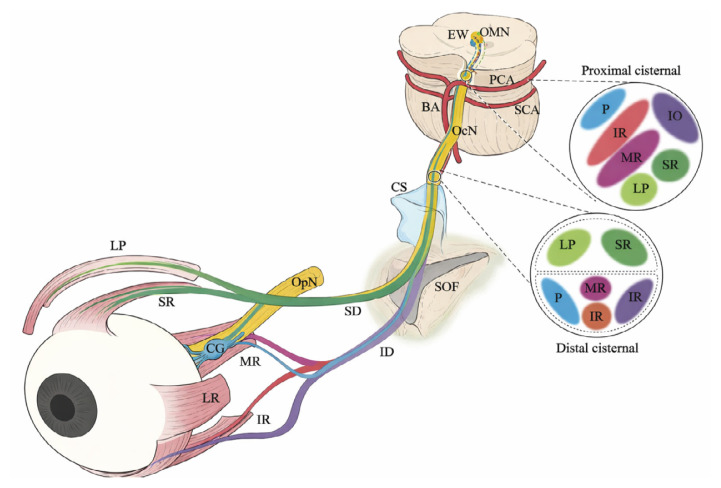
Image showing the course of the oculomotor nerve and location of the parasympathetic segment in the nerve throughout its course

Preganglionic parasympathetic neurons originating in the EW nuclei exit the midbrain within the oculomotor nerve. The intracranial course of the oculomotor nerve, from its emergence in the interpeduncular fossa to entry into the cavernous sinus, measures approximately 15 to 20 mm, based on cadaveric microsurgical series. Parasympathetic fibres are located superficially within the oculomotor nerve and travel predominantly in its inferior division after passage through the superior orbital fissure, before synapsing in the ciliary ganglion, a parasympathetic ganglion measuring approximately 2 to 3 mm in diameter and located posterolateral to the optic nerve within the orbit (Figure 4) (2, 5).

**Figure 5:**
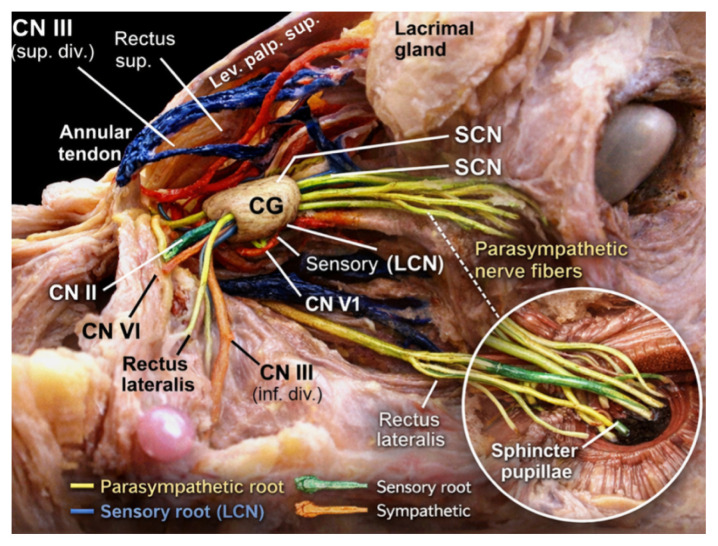
Parasympathetic supply to the pupils via the ciliary ganglion

Postganglionic fibres arise from the ciliary ganglion and course via the short ciliary nerves, traversing the sclera to innervate the sphincter pupillae muscle of the iris. Acetylcholine acting on muscarinic (M3) receptors mediates contraction of the sphincter pupillae, resulting in pupillary constriction (miosis). While the intraocular course of the short ciliary nerves is short and variable, their terminal distribution within the iris stroma is consistently demonstrated in human histopathological and experimental neuroanatomical studies (Figure 5) (3).

### Sympathetic Pathway Regulating Pupillary Dilation

#### First-order (Central) Sympathetic Neuron

**Figure 6:**
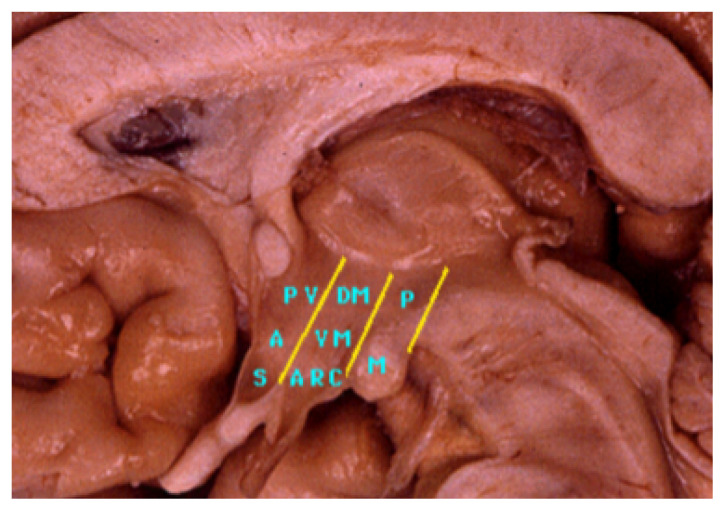
The hypothalamus is organised into anterior, middle (tuberal), and posterior regions, each containing the principal nuclei relevant to its function The anterior region includes the suprachiasmatic and supraoptic nuclei (S), as well as the anterior hypothalamic nucleus (A) and the paraventricular nucleus (PV); The middle (tuberal) region contains the arcuate nucleus (ARC), ventromedial nucleus (VM), and dorsomedial nucleus (DM); The posterior region is characterised by the posterior hypothalamic nucleus (P) and the mammillary nuclei (M)

**Figure 7:**
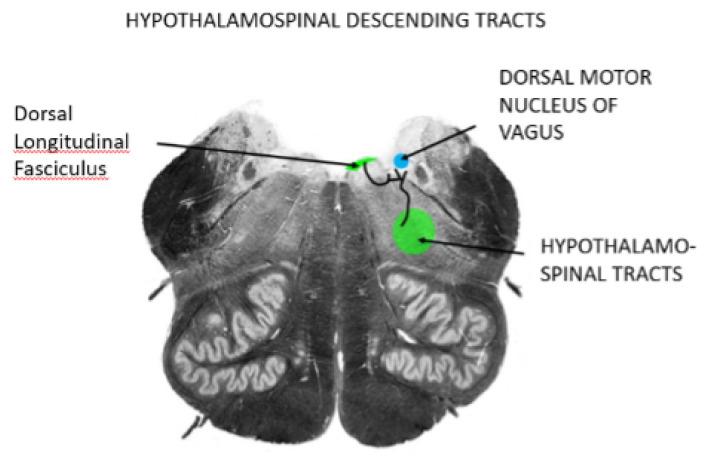
Cross-section showing the location of hypothalamospinal tracts

**Figure 8:**
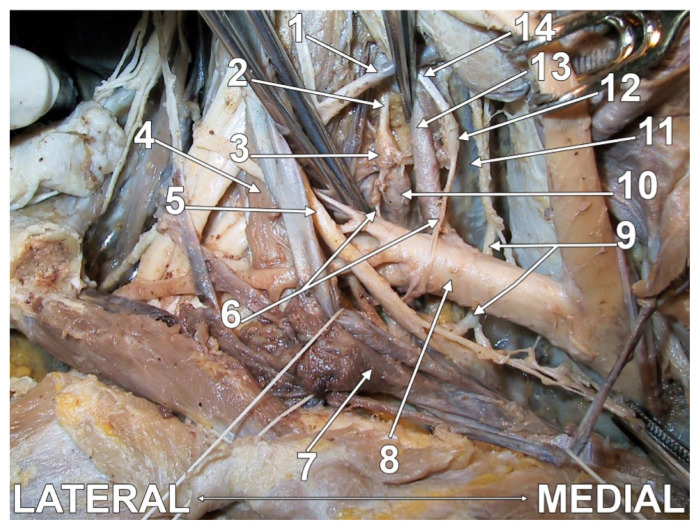
Original dissection of the inferior cervical and superior thoracic ganglia—incomplete stellate ganglion formation, and ansa subclavia Anterior view. Right side: 1 = inferior thyroid artery; 2 = vertebral nerve; 3 = inferior cervical ganglion; 4 = anterior scalene muscle; 5 = vagus nerve; 6 = ansa subclavia; 7 = brachiocephalic (innominate) vein; 8 = subclavian artery; 9 = recurrent laryngeal nerve; 10 = T1 sympathetic ganglion; 11 = longus colli muscle; 12= intermediate (vertebral) ganglion; 13 = vertebral artery; 14 = cervical sympathetic trunk; Reproduced from Rusu et al. (5)

The first-order sympathetic neurons mediating pupillary dilation originate in the posterolateral hypothalamus (Figure 6) (2, 4). Axons descend ipsilaterally through the diencephalon and brainstem as the hypothalamospinal tract, coursing through the lateral tegmentum of the midbrain, pons, and medulla without synapsing (Figure 7) (2, 4). These fibres terminate in the ciliospinal centre of Budge, located in the intermediolateral cell column of the spinal cord at levels C8–T2, most prominently at T1 (Figure 8) (2, 4). The descending hypothalamospinal tract spans the rostrocaudal extent from the hypothalamus to the upper thoracic spinal cord, with an estimated length of approximately 20 to 30 cm, depending on individual body habitus and vertebral level of termination. Lesions involving this segment result in a central (first-order) Horner syndrome (2).

#### Second-order (Preganglionic) Sympathetic Neuron

**Figure 9:**
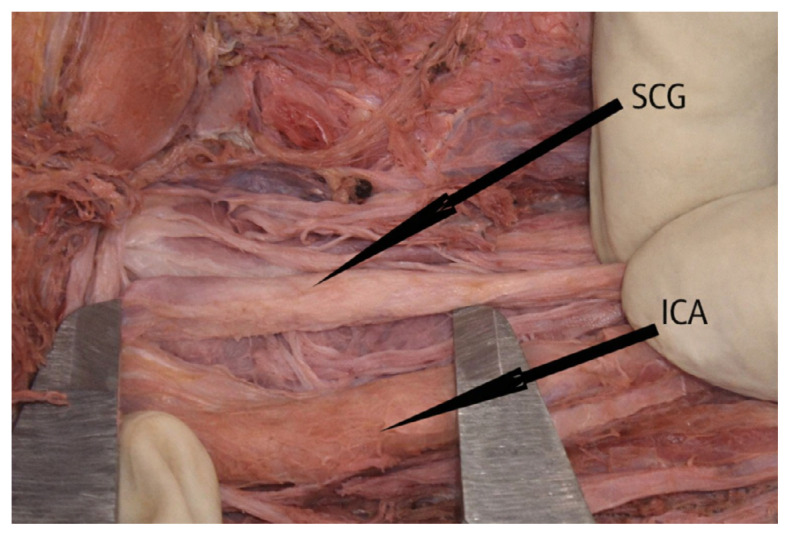
Superior cervical ganglion (SCG) and internal carotid artery (ICA)

Second-order preganglionic sympathetic neurons arise from the intermediolateral cell column of the upper thoracic spinal cord, principally at the T1 level. Their axons exit the spinal cord via the ventral roots, enter the sympathetic chain through the white rami communicantes, and ascend within the cervical sympathetic trunk. These fibres pass superiorly over the lung apex and synapse in the superior cervical ganglion, typically located at the C2–C3 vertebral level near the carotid bifurcation (Figure 9) (2, 4). The preganglionic segment extends from the upper thoracic spinal cord to the superior cervical ganglion and measures approximately 15 to 20 cm in length. Interruption of this segment results in a preganglionic Horner syndrome and is classically associated with lesions of the lung apex, neck, or thoracic outlet (2).

#### Third-order (Postganglionic) Sympathetic Neuron

**Figure 10:**
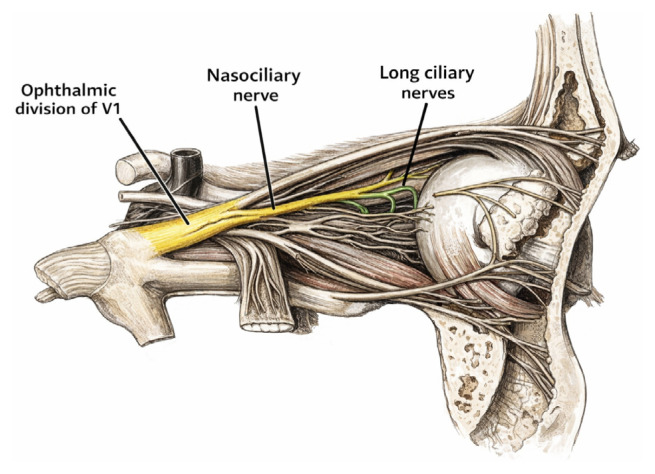
Sympathetic supply of the dilator pupillae muscle by the long ciliary nerve

Third-order sympathetic neurons arise from the superior cervical ganglion, typically located opposite the C2–C3 vertebral levels. Postganglionic fibres form the internal carotid nerve and ascend along the cervical segment of the internal carotid artery, constituting the internal carotid sympathetic plexus. In terms of Bouthillier’s classification, these fibres initially accompany the cervical segment (C1) of the internal carotid artery in the neck, corresponding approximately to the C2–C4 vertebral levels (2, 6). The sympathetic plexus then continues intracranially with the internal carotid artery through the petrous (C2) and cavernous (C4) segments, where sympathetic fibres distribute to the cavernous sinus and subsequently join branches of the ophthalmic division of the trigeminal nerve. Terminal fibres enter the orbit via the superior orbital fissure to innervate the iris dilator pupillae muscle and Müller’s (superior tarsal) muscle (Figure 10) (2, 6).

The postganglionic segment, extending from the superior cervical ganglion to the orbital structures, measures approximately 10 to 15 cm in length. Lesions affecting this segment result in postganglionic Horner syndrome and are characteristically associated with preserved facial sweating when the lesion lies distal to the carotid bifurcation (2).

### Physiological Pupillary Constriction and Dilation (Wellness)

Miosis and mydriasis are fundamentally physiological phenomena, reflecting the dynamic balance between parasympathetic and sympathetic influences on the iris musculature (2, 3). In healthy individuals, miosis refers to pupillary constriction that occurs predominantly in response to increased ambient illumination, mediated by activation of the parasympathetic pathway originating in the midbrain (2, 3). Conversely, mydriasis denotes pupillary dilation under conditions of reduced illumination, darkness, or heightened arousal, mediated by sympathetic activation of the iris dilator muscle (2, 3). In this physiological context, pupillary size is bilaterally symmetric, reactive, and appropriately modulated by environmental light levels and cognitive state, serving to optimise retinal illumination and visual acuity (3).

### Pathological Pupillary Constriction and Dilation (Illness)

In contrast, miosis and mydriasis in illness represent disordered pupillary states arising from dysfunction of central or peripheral components of the autonomic or brainstem pathways governing pupillary control (2, 7). Pathological mydriasis may occur due to impairment of parasympathetic outflow at the level of the midbrain, oculomotor nerve, or ciliary ganglion, or from compressive or ischaemic processes affecting these structures (2, 7). Similarly, pathological miosis may result from disruption of the sympathetic pathway to the eye, leading to unopposed parasympathetic tone, or from intrinsic brainstem or pharmacologic influences (2, 9). In disease states, pupillary abnormalities are often asymmetric, poorly reactive, or fixed, and their presence reflects underlying structural or functional compromise rather than adaptive physiological regulation (7).

### Conceptual Distinction (Key Principle)

**Figure 11:**
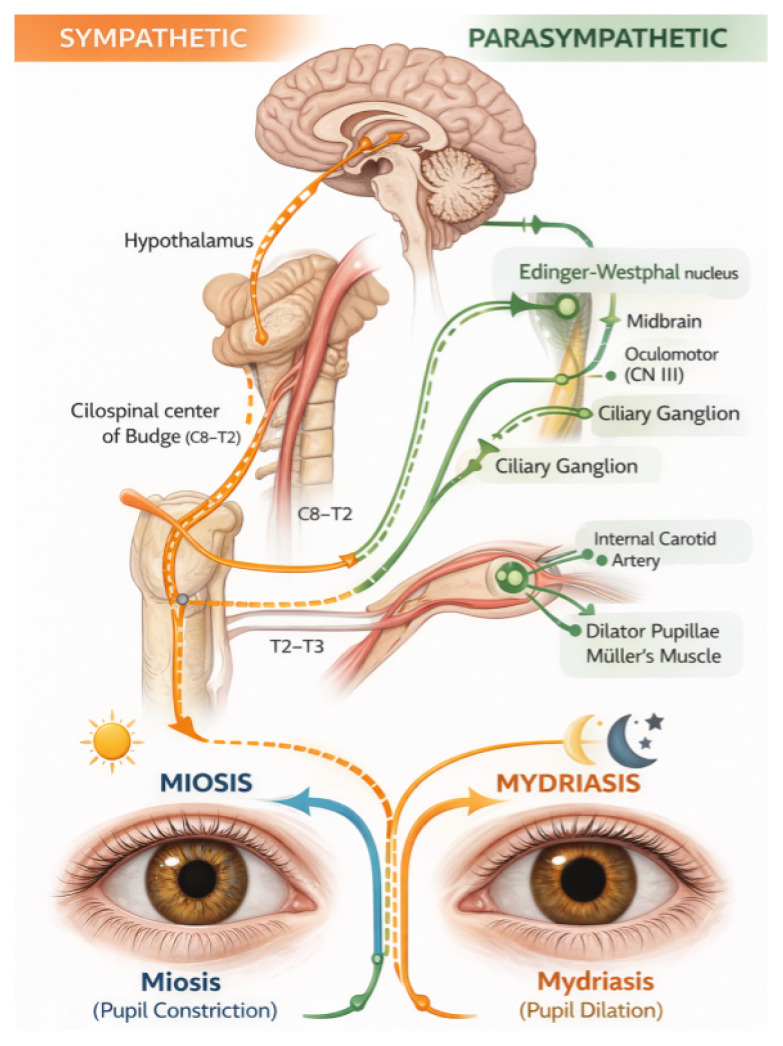
Sympathetic and parasympathetic pathways regulating pupillary diameter

The critical distinction between physiological and pathological pupillary changes lies not in the direction of pupillary size change itself, but in the context, symmetry, and reactivity of the response (2, 7). While miosis and mydriasis are normal adaptive responses to environmental and behavioural stimuli, their occurrence in illness signifies a breakdown in the integrity of brainstem or autonomic control mechanisms. As such, pupillary examination provides a valuable clinical window into the functional status of these neural systems (Figure 11) (2, 7).

## Herniation Syndromes and Correlation to Pupillary Size with Localisation of Lesion

### Central Herniation

#### Diencephalic Stage

In the diencephalic stage of downward transtentorial herniation, pupillary examination typically reveals small pupils measuring approximately 1 to 3 mm that remain reactive to light, though with a limited range of excursion (1, 7). Compression involves the posterior hypothalamus and upper diencephalon above the midbrain, while intrinsic brainstem nuclei remain structurally intact (1, 7). At this stage, the descending hypothalamospinal sympathetic fibres are disrupted early. These fibres are characteristically long, poorly myelinated, and highly vulnerable to stretch and ischaemia, leading to early loss of sympathetic pupillary tone (2, 7).

In contrast, parasympathetic pathways remain functional, as the Edinger–Westphal nuclei and oculomotor fascicles are not yet compressed. Consequently, parasympathetic influence becomes relatively unopposed, resulting in small pupils. Maximal constriction does not occur, however, as some cortical and supranuclear modulation is still preserved, explaining the combination of small size, retained reactivity, and limited excursion (2, 7). Loss of sympathetic tone alone therefore produces small but reactive pupils (Table 1) (2).

#### Midbrain (Upper Pontine) Stage

Progression of herniation to the level of the tentorial notch results in compression of the midbrain tegmentum, including the Edinger– Westphal nuclei and oculomotor nerve fascicles (1, 7). Clinically, this stage is characterised by bilaterally mid-position pupils, typically 3 to 5 mm in diameter, that are fixed and nonreactive (7, 8). Sympathetic pupillary pathways have already been disrupted during the earlier diencephalic stage, and parasympathetic function is now abolished due to ischaemic injury of the Edinger–Westphal nuclei and compression of parasympathetic fibres within the oculomotor nerve (Table 1) (7, 8).

With both autonomic systems rendered nonfunctional, the iris musculature assumes its mechanical resting position. This anatomic midpoint corresponds to a pupillary diameter of approximately 3 to 5 mm. Mid-position fixed pupils therefore localise precisely to midbrain failure and represent a hallmark of central herniation, rather than pharmacologic dilation or uncal herniation (7, 8).

#### Lower Pontine Stage (Transitional)

Although often described as a distinct stage, classic pinpoint pupils are uncommon during downward central herniation. By the time herniation reaches the lower pons, parasympathetic pathways have already been destroyed at the midbrain level (7). As a result, the selective parasympathetic preservation required to produce pinpoint pupils is absent. True pinpoint pupils are therefore more characteristic of intrinsic pontine lesions, such as pontine haemorrhage, in which descending sympathetic pathways are interrupted while parasympathetic nuclei remain intact (7, 9).

In contrast, herniation produces combined ischaemic and stretch injury, leading to nonselective destruction of both autonomic systems and precluding sustained pinpoint pupillary responses (Table 1) (7).

#### Medullary Stage (Terminal)

In the terminal medullary stage of herniation, pupils become bilaterally dilated and fixed, typically measuring approximately 7 to 9 mm (7, 8). At this point, parasympathetic function has long been abolished due to infarction of the Edinger–Westphal nuclei and failure of the oculomotor nerves. Sympathetic pathways are also nonfunctional, having been disrupted earlier at their hypothalamic origin and along the cervicothoracic outflow (2, 7).

Despite the absence of sympathetic activity, the pupils dilate maximally due to complete autonomic denervation of the iris. This results in iris muscle atony, whereby both sphincter and dilator muscles lose neural tone, allowing the pupil to assume its maximal passive diameter. This dilation reflects elastic recoil of the iris stroma and mechanical dominance of the dilator architecture rather than sympathetic overactivity (7, 8). Such bilaterally blown pupils signify terminal brainstem failure and are analogous to pupillary findings in brain death or profound global anoxic injury (Table 1) (7, 8).

## Principles of Neurosurgical Intervention Across Stages of Rostrocaudal Herniation

Rostrocaudal brainstem herniation represents a dynamic, rapidly evolving process rather than a fixed, stepwise cascade (7, 8). Although classically described in diencephalic, midbrain/upper pontine, lower pontine, and medullary stages, clinical transition between stages may occur within minutes and does not necessarily follow a predictable or linear sequence (7). Pupillary findings reflect progressive dysfunction of parasympathetic and sympathetic pathways but may lag behind irreversible neuronal injury (7, 8).

Consequently, neurosurgical intervention is guided by physiological reversibility, timing, and global neurological context rather than pupillary size alone. Because progression between herniation stages may be rapid and non-sequential, management decisions should be informed by overall clinical status, neuroimaging findings, and temporal evolution rather than isolated pupillary changes (Table 2) (7–9).

### Diencephalic Stage: Reversal-directed Intervention

During the diencephalic stage of rostrocaudal herniation, neurosurgical management is directed toward rapid reversal of intracranial hypertension prior to midbrain involvement (7, 8). Neurological dysfunction at this level is potentially reversible, and early intervention is indicated (7). Medical management consists of immediate intracranial pressure reduction and optimisation of cerebral perfusion, including hyperosmolar therapy, head elevation, sedation, controlled ventilation, and hemodynamic support (8). Surgical management is pursued when a surgically remediable cause is identified and includes urgent evacuation of mass lesions, cerebrospinal fluid diversion, or decompressive craniectomy, guided by imaging findings and clinical trajectory (8). This approach reflects established principles describing the diencephalic stage as a therapeutic window during which neurological deterioration may still be reversible (7, 8).

### Upper Pontine Stage: Time-limited Salvage Intervention

The upper pontine stage reflects direct compression of the midbrain, with impairment of consciousness and pupillary reactivity (7, 8). At this stage, neurological injury is often advanced; however, intervention may be considered in selected cases, particularly when deterioration is acute and the underlying cause is surgically reversible (8). Medical management involves maximal intracranial pressure–lowering measures, with hyperventilation and vasoactive support used as short-term stabilising strategies rather than definitive therapy (8). Surgical management, when undertaken, is salvage-oriented, targeting rapid decompression of a causative lesion with acknowledgement of a guarded neurological prognosis (8). This stage represents a transition point beyond which the likelihood of neurological recovery declines sharply, necessitating individualised decision-making (7, 8).

### Lower Pontine Stage: Limited Therapeutic Utility

The lower pontine stage indicates extensive brainstem involvement, including disruption of descending sympathetic pathways and respiratory centres, and is associated with a very low probability of meaningful neurological recovery (7, 9). Medical management is restricted to supportive care, including airway protection, ventilatory support, and circulatory stabilisation, with recognition that intracranial pressure–lowering measures are unlikely to alter outcome (8, 9). Surgical management is generally not indicated, as decompressive intervention at this stage rarely improves neurological prognosis and may only prolong physiological survival (8, 9).

### Medullary Stage: Futility of Intervention

The medullary stage represents terminal rostrocaudal herniation, characterised by failure of cardiorespiratory centres and global autonomic collapse. Neurological injury at this stage is irreversible (7, 9). Medical management is limited to supportive or comfort-directed care (9). Surgical management has no therapeutic role, as decompression does not restore medullary function or alter neurological outcome (7, 9). This stage is uniformly described as incompatible with neurological recovery (7, 9).

## Figures and Tables

**Table 1:** Pupillary characteristics and autonomic correlates across stages of rostrocaudal brainstem herniation (1, 2, 7)

Herniation stage	Typical pupil size	Pupillary reactivity	Dominant anatomical level	Key autonomic pathology	Core mechanism
Diencephalic stage	Small (1 to 3 mm)	Reactive, limited excursion	Posterior hypothalamus/upper diencephalon	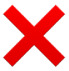 Sympathetic 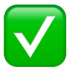 Parasympathetic preserved	Early disruption of hypothalamospinal fibres→ unopposed parasympathetic tone
Midbrain (upper pontine) stage	Mid-position (3 to5 mm)	Fixed, nonreactive	Midbrain tegmentum (tentorial notch)	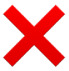 Sympathetic 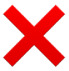 Parasympathetic	Loss of both autonomic inputs → iris assumes mechanical midpoint
Lower pontine stage (transitional)	Mid-position (3 to 5 mm) Variable; pinpoint uncommon	Usually nonreactive	Lower pons (stretch/ischaemia)	Both systems already compromised	Pinpoint pupils require selective sympathetic loss → not typical of herniation
Medullary (terminal) stage	Dilated (7 to 9 mm)	Fixed	Medulla/global brainstem	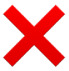 Sympathetic 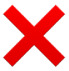 Parasympathetic	Complete autonomic denervation → iris muscle atony and maximal passive dilation

**Table 2:** Stage-based medical and neurosurgical management of rostrocaudal brainstem herniation (7–9)

Stage	Medical management	Neurosurgical management
Diencephalic	ICP reduction (hyperosmolar therapy), sedation, ventilation, CPP optimisation	Urgent mass lesion evacuation, CSF diversion, decompressive craniectomy
Upper pontine (midbrain)	Maximal ICP therapy; short-term hyperventilation as bridge	Selective salvage decompression for reversible causes
Lower pontine	Supportive care only (airway, ventilation, hemodynamics)	Generally not indicated
Medullary	Supportive or comfort-directed care	No role for surgical intervention
